# Development of a clinical prediction model for intra-abdominal infection in severe acute pancreatitis using logistic regression and nomogram

**DOI:** 10.3389/fmed.2025.1636733

**Published:** 2025-08-07

**Authors:** Rui Qi, He-Bin Wang, Ren-Ying Luo, Jing Li, Li Su

**Affiliations:** ^1^Department of Hepatology, Panzhihua Hospital of Integrated Chinese and Western Medicine, Panzhihua, China; ^2^Department of Intensive Care Medicine, Panzhihua Hospital of Integrated Chinese and Western Medicine, Panzhihua, China

**Keywords:** acute pancreatitis, severe acute pancreatitis, intra-abdominal infection, nomogram, decision curve analysis, predictive model

## Abstract

**Objective:**

This study aimed to develop and validate a clinical prediction model for identifying intra-abdominal infection (IAI) in patients with severe acute pancreatitis (SAP).

**Methods:**

We conducted a retrospective cohort study of patients diagnosed with SAP at our institution between January 2020 and December 2023. A total of 415 eligible patients were enrolled and randomly allocated into a training set (*n* = 291) and a validation set (*n* = 124) in a 7:3 ratio for model development and internal validation. In the training cohort, candidate predictors were selected using least absolute shrinkage and selection operator (LASSO) regression to mitigate overfitting and retain the most clinically relevant variables. A multivariable logistic regression model was subsequently constructed, and a nomogram was developed to facilitate individualized risk assessment. Model performance was evaluated based on discrimination, calibration, and clinical utility. Discrimination was assessed using the area under the receiver operating characteristic curve (AUC) in both cohorts. Calibration was examined via calibration plots with bootstrapping (1,000 resamples) to correct for optimism. Decision curve analysis (DCA) was performed to determine the net clinical benefit across different risk thresholds.

**Results:**

The final cohort comprised 415 patients, with 291 in the training set and 124 in the validation set. LASSO regression identified four independent predictors with non-zero coefficients: hematocrit (HCT), procalcitonin (PCT), Acute Physiology and Chronic Health Evaluation II (APACHE II) score, and neutrophil-to-lymphocyte ratio (NLR). The prediction model demonstrated robust discrimination, with an AUC of 0.853 (95% CI: 0.804–0.901) in the training set and 0.858 (95% CI: 0.786–0.930) in the validation set. Calibration plots indicated excellent agreement between predicted and observed probabilities. DCA confirmed significant clinical utility across a wide range of risk thresholds.

**Conclusion:**

The proposed prediction model, incorporating HCT, PCT, APACHE II, and NLR, accurately stratifies the risk of IAI in SAP patients. This tool may facilitate early risk identification, guide timely antibiotic therapy, and optimize clinical decision-making to improve patient outcomes.

## 1 Introduction

Severe acute pancreatitis (SAP) represents a life-threatening manifestation of acute pancreatitis, defined by persistent organ failure and associated with substantial mortality, particularly when complicated by intra-abdominal infection (IAI). IAI is a key determinant of adverse outcomes in SAP, with reported mortality rates escalating to 47–69% in cases complicated by secondary sepsis, multi-organ dysfunction syndrome (MODS), or intra-abdominal hypertension (IAH) (1).

The early identification of SAP patients at high risk for IAI is critical for implementing timely antimicrobial therapy, intensified monitoring, and targeted interventions to mitigate disease progression. However, existing predictive strategies remain suboptimal. While systemic inflammatory markers—including C-reactive protein (CRP), interleukin-6 (IL-6), and procalcitonin (PCT)—demonstrate sensitivity to infection, their specificity for IAI in SAP is limited, as they also reflect sterile inflammation and pancreatic necrosis (2–4). Similarly, hematologic indices such as hematocrit (HCT), platelet count (PLT), and neutrophil-to-lymphocyte ratio (NLR) correlate with systemic inflammation and disease severity but lack discriminatory power for IAI prediction (5, 6).

Although established scoring systems like the Acute Physiology and Chronic Health Evaluation II (APACHE II) and radiographic classifications such as the Computed Tomography Severity Index (CTSI) aid in overall SAP risk stratification, their utility in specifically forecasting IAI remains inadequate (7, 8). Despite advances in biomarker research, no validated, clinically practical tool currently exists for individualized IAI risk assessment in SAP.

To address this unmet need, we conducted a comprehensive evaluation of clinical, laboratory, and scoring-based predictors to develop and validate a multivariable prediction model for early IAI detection in SAP. This model aims to enhance risk stratification, optimize therapeutic decision-making, and ultimately improve patient outcomes through timely intervention.

## 2 Methods

### 2.1 Study population

This retrospective study included patients diagnosed with SAP at our institution between January 2020 and December 2023. Baseline demographic and clinical data, including age and sex, were collected. The study protocol was approved by the Panzhihua Hospital of Integrated Chinese and Western Medicine ethics committee (Approval No. 2022-10-027).

### 2.2 Inclusion and exclusion criteria

**Inclusion criteria:**

1. Age between 18 and 80 years;
2. Diagnosis of SAP according to established classification criteria for acute pancreatitis;
3. Symptom onset to hospital admission occurring within 72 h;
4. Availability of comprehensive clinical data, including PLT, HCT, CRP, IL-6, and NLR.

**Exclusion criteria:**

1. Patients with malignant tumors, chronic organ dysfunction, or a history of other severe comorbidities;
2. Patients presenting with gastrointestinal bleeding, intestinal obstruction, or other significant gastrointestinal disorders;
3. Pregnant individuals, patients with autoimmune diseases, those with traumatic SAP, and patients with fulminant acute pancreatitis;
4. Patients who underwent open abdominal surgery before or during hospitalization.

### 2.3 Diagnostic criteria

IAI was diagnosed based on positive microbiological cultures (bacterial or fungal) obtained from sterile percutaneous or intraoperative aspirates of peritoneal fluid, pancreatic necrosis, or peripancreatic collections. Systemic Inflammatory Response Syndrome (SIRS) was defined according to established clinical criteria, requiring the presence of at least two of the following: (1) core body temperature >38°C or <36°C; (2) respiratory dysfunction evidenced by arterial pCO₂ < 32 mmHg or respiratory rate >20 breaths/min or clinical signs of hyperventilation; (3) tachycardia with heart rate >90 beats/min; (4) leukocyte abnormalities including leukocytosis (>12 × 10^9^/L), leukopenia (<4 × 10^9^/L), or presence of >10% immature neutrophils (band forms).

### 2.4 Study variables and data collection

Demographic, clinical, and laboratory parameters were systematically collected, including: sex, age, comorbidities (hypertension, diabetes mellitus), lifestyle factors (tobacco use, alcohol consumption), SAP etiology, body mass index (BMI), and inflammatory biomarkers (neutrophil count [NEUT], PLT, HCT, D-dimer, CRP, IL-6, PCT). Disease severity was assessed using the Systemic Inflammatory Response Syndrome (SIRS) criteria and APACHE II score, while the NLR served as an additional inflammatory marker.

All laboratory parameters were obtained during the initial 24 h of hospitalization, preceding any surgical procedures or antimicrobial therapy initiation. These admission values were utilized for predictive modeling to ensure temporal consistency. For patients with repeated measurements within the first 48 h, only the initial values were incorporated to maintain data uniformity and prevent confounding by treatment effects.

IAI status was definitively determined through microbiological confirmation during the hospitalization period, serving as the primary outcome measure.

### 2.5 Statistical power considerations and overfitting mitigation

The study cohort (*N* = 415) was randomly partitioned into a derivation set (*n* = 291, 70%) and an internal validation set (*n* = 124, 30%) to facilitate model development and performance evaluation. To enhance model stability and minimize overfitting, we implemented bootstrap resampling with 1,000 iterations during the model-building phase.

The initial model incorporated 18 candidate predictor variables, necessitating careful consideration of the event-per-variable (EPV) ratio. Current methodological standards recommend maintaining an EPV ≥ 10 to ensure model reliability and generalizability. In our derivation cohort, 90 IAI events were observed, yielding an EPV of 5.0 (90 events/18 predictors). While this ratio falls below conventional recommendations, we employed rigorous statistical techniques (including bootstrap validation and shrinkage methods) to mitigate potential overfitting and optimize model performance.

### 2.6 Statistical analysis

All statistical analyses were performed using R version 4.2.2 and MSTATA.^1^ A two-sided *p* value < 0.05 was considered statistically significant.

Continuous variables were expressed as median and interquartile range [M (P25, P75)], and compared using the Wilcoxon rank-sum test due to non-normal distribution. Categorical variables were described as counts and percentages (n, %) and compared using the Chi-square test or Fisher’s exact test, as appropriate.

To identify independent predictors of IAI in SAP, we applied Least Absolute Shrinkage and Selection Operator (LASSO) logistic regression on the training set. A total of 18 clinically relevant variables were initially entered. The optimal penalization coefficient (λ) was determined by 10-fold cross-validation, selecting the λ corresponding to the minimum mean squared error (MSE). Based on LASSO selection, four predictors with non-zero coefficients were retained, these variables were subsequently entered into a multivariable logistic regression model, and the final model equation was:

log[ $\hat{P}(1-\hat{P})$ ] = 9.718–0.319 (HCT) + 0.705 (PCT) − 0.11 (NLR) + 0.174 (APACHE-II). Model performance was assessed via: receiver Operating Characteristic (ROC) analysis in both training and validation cohorts, yielding AUCs of 0.853 and 0.858, respectively; calibration curves comparing predicted vs. observed risk; decision Curve Analysis (DCA) evaluating net clinical benefit across threshold probabilities.

To mitigate overfitting and quantify optimism, bootstrap resampling (1,000 iterations) was conducted, yielding an optimism-adjusted AUC of 0.832. A nomogram was constructed to facilitate clinical use, and individual predictor diagnostic value was further visualized via ROC curves.

**Figure 1:**
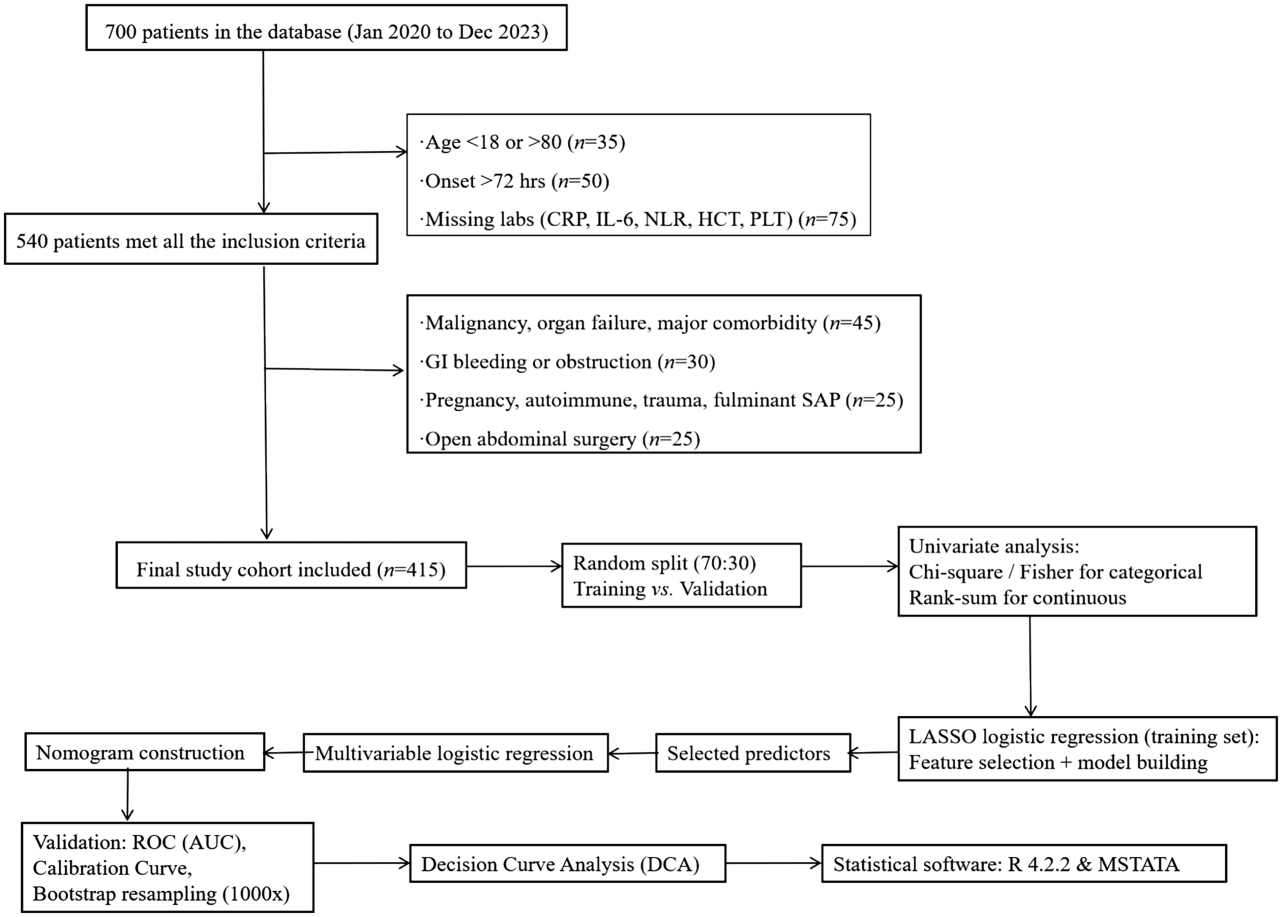
The flowcharts for data filtering. APACHE II score was recorded as an observational variable, not used for exclusion.

The flowcharts for data filtering are shown in Figure 1.

## 3 Results

### 3.1 Demographic and clinical characteristics

The prediction analysis included 415 participants with baseline clinical and demographic characteristics. The cohort was stratified into a training set (*n* = 291) and an internal validation set (*n* = 124). Comparative analysis demonstrated balanced distributions of clinical features and demographic variables between the two cohorts (Table 1).

**Table 1:** Demographic and clinical characteristics of the training and test cohorts.

Characteristic	Overall (*N* = 415)	Training (*N* = 291)	Validation (*N* = 124)	*p*-value
Sex - Female, *n* (%)	236 (56.9%)	160 (55.0%)	76 (61.3%)	0.235
Sex - Male, *n* (%)	179 (43.1%)	131 (45.0%)	48 (38.7%)	
Age, years [M (P_25_, P_75_)]	48.0 [45.0–51.5]	48.0 [44.0–51.5]	48.0 [45.0–51.3]	
History of smoking - No, *n* (%)	298 (71.8%)	204 (70.1%)	94 (75.8%)	0.237
History of smoking - Yes, *n* (%)	117 (28.2%)	87 (29.9%)	30 (24.2%)	
Alcohol consumption - No, *n* (%)	297 (71.6%)	208 (71.5%)	89 (71.8%)	0.951
Alcohol consumption - Yes, *n* (%)	118 (28.4%)	83 (28.5%)	35 (28.2%)	
History of hypertension - No, *n* (%)	323 (77.8%)	229 (78.7%)	94 (75.8%)	0.517
History of hypertension - Yes, *n* (%)	92 (22.2%)	62 (21.3%)	30 (24.2%)	
History of diabetes - No, *n* (%)	371 (89.4%)	259 (89.0%)	112 (90.3%)	0.689
History of diabetes - Yes, *n* (%)	44 (10.6%)	32 (11.0%)	12 (9.7%)	
Etiology - Alcoholic, *n* (%)	64 (15.4%)	43 (14.8%)	21 (16.9%)	0.18
Etiology - Biliary, *n* (%)	167 (40.2%)	118 (40.5%)	49 (39.5%)	
Etiology - Hypertriglyceridemia, *n* (%)	137 (33.0%)	91 (31.3%)	46 (37.1%)	
Etiology - Others, *n* (%)	47 (11.3%)	39 (13.4%)	8 (6.5%)	
BMI [M (P_25_, P_75_)]	25.0 [22.0–28.0]	25.0 [22.0–28.0]	24.0 [21.0–27.0]	0.019
NEUT [M (P_25_, P_75_)]	86.5 [84.5–88.6]	86.5 [84.6–88.6]	86.2 [84.3–88.4]	0.401
PLT [M (P_25_, P_75_)]	164 [142–187]	161 [142–185]	168 [145–190]	0.165
NLR [M (P_25_, P_75_)]	5.3 [2.8–9.0]	6.0 [2.9–9.5]	5.0 [2.4–8.0]	0.106
HCT[M (P_25_, P_75_)]	38.2 [34.3–40.3]	38.3 [34.3–40.4]	37.9 [34.4–40.2]	0.447
D-dimer [M (P_25_, P_75_)]	2.34 [1.38–3.18]	2.35 [1.42–3.21]	2.34 [1.22–3.02]	0.34
CRP [M (P_25_, P_75_)]	133 [97–169]	130 [96–165]	135 [106–176]	0.151
IL-6 [M (P_25_, P_75_)]	55 [30–82]	53 [30–78]	59 [31–87]	0.35
PCT [M (P_25_, P_75_)]	1.19 [0.72–1.74]	1.23 [0.77–1.70]	1.09 [0.68–1.79]	0.671
SIRS - No, *n* (%)	124 (29.9%)	86 (29.6%)	38 (30.6%)	0.824
SIRS - Yes, *n* (%)	291 (70.1%)	205 (70.4%)	86 (69.4%)	
APACHE II score [M (P_25_, P_75_)]	9.00 [8.00–11.00]	9.00 [8.00–11.00]	9.00 [8.00–10.25]	0.444

### 3.2 Diagnostic factor selection and multicollinearity assessment

Eighteen candidate predictors were initially evaluated based on clinical relevance and admission availability: sex, age, smoking history, alcohol consumption, hypertension, diabetes, SAP etiology, BMI, NEUT, PLT, HCT, D-dimer, CRP, IL-6, PCT, SIRS, APACHE II score, and NLR. Dimensionality reduction was performed using LASSO regression with 10-fold cross-validation in the training cohort.

**Figure 2:**
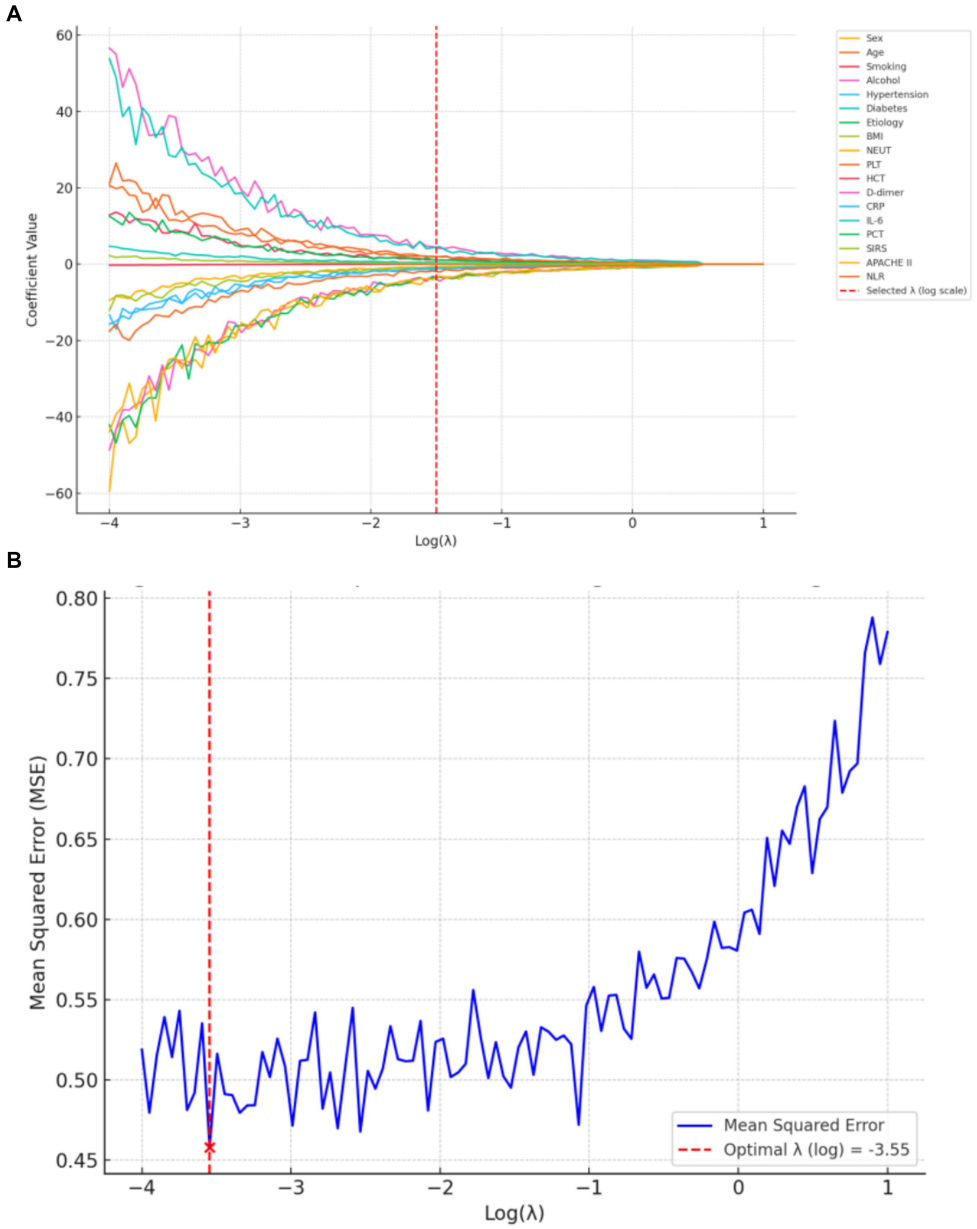
LASSO regression for predictor selection. **(A)** Shrinkage trajectories of regression coefficients versus Log(λ). **(B)** 10-fold cross-validation plot for LASSO: mean squared error (MSE) plotted against Log(λ). The optimal λ (0.035) was selected at the point of minimum MSE.

**Figure 3:**
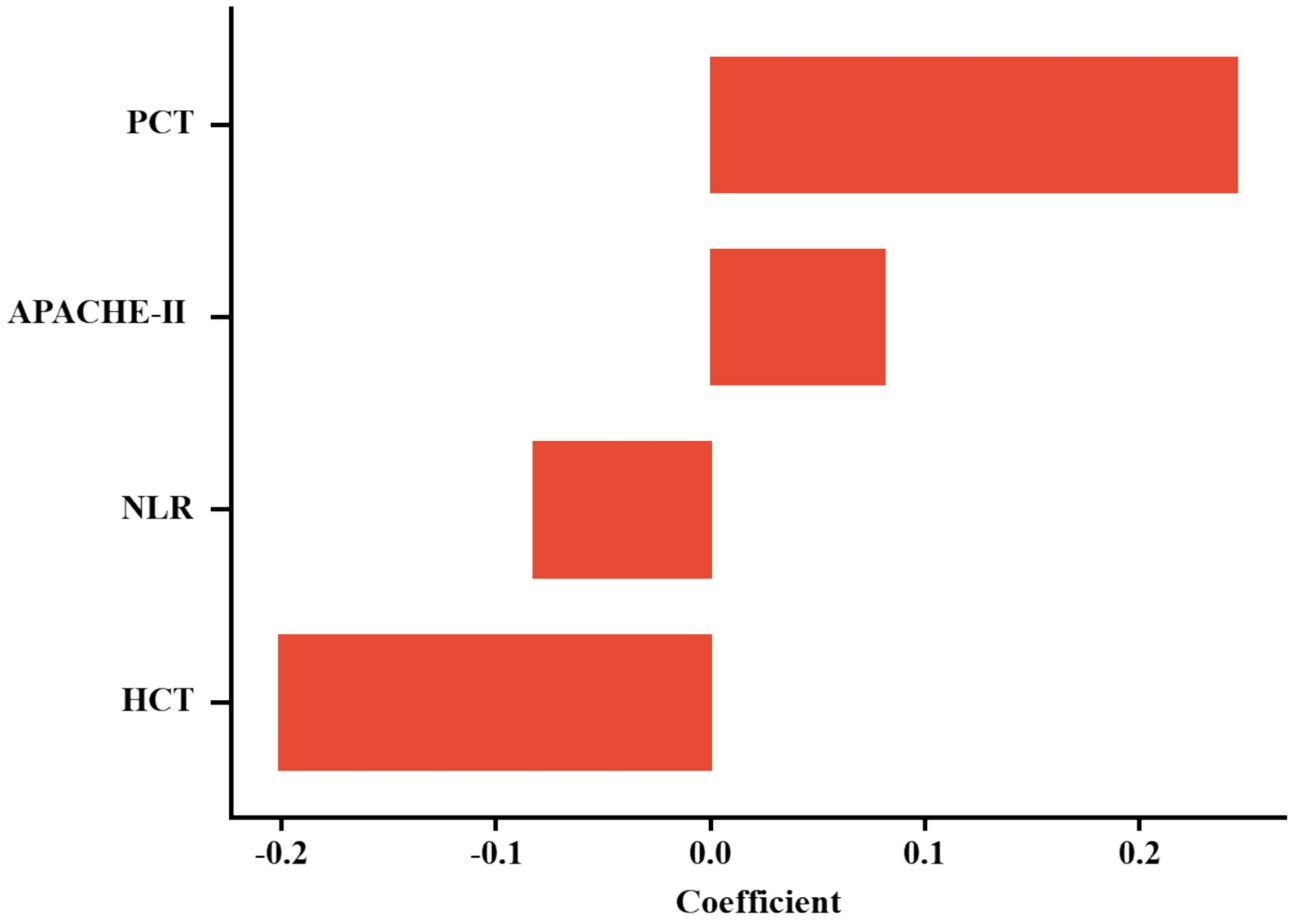
Histogram of non-zero coefficients from LASSO regression.

The optimal regularization parameter (λ = 0.035) was determined through minimum mean cross-validated error (min-MSE) criteria (Figures 2A,B). Feature selection identified four non-zero coefficient predictors: HCT, PCT, APACHE II score, and NLR (Figure 3). Notably, established inflammatory markers (CRP, IL-6) were excluded during LASSO penalization, likely due to multicollinearity with integrative predictors (NLR, APACHE II) and limited incremental predictive value.

**Figure 4:**
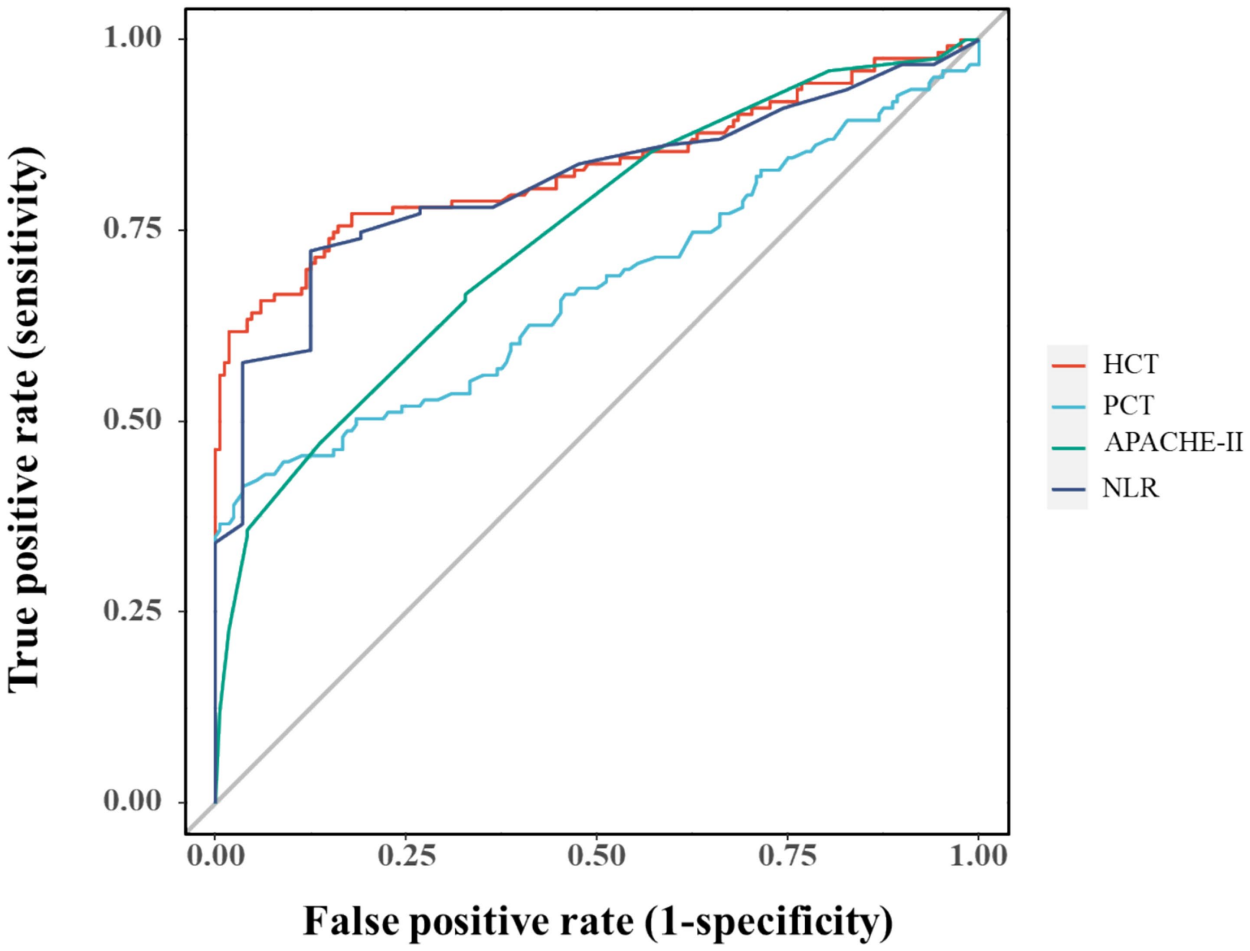
ROC curves for individual variables diagnosing IAI.

Multicollinearity assessment via VIF confirmed acceptable levels (all <2, Supplementary Table S1). Individual predictor performance was quantified through ROC analysis (Figure 4; Table 2). Multivariate logistic regression revealed inverse associations between HCT/NLR and IAI risk (OR < 1), potentially reflecting microcirculatory optimization or immune regulation, while APACHE II demonstrated borderline significance (*p* = 0.088) (Table 3).

**Table 2:** AUC values and 95% CI for variables.

Variable	AUC	95% CI
HCT	0.833	(0.781–0.886)
PCT	0.675	(0.609–0.741)
APACHE-II scores	0.744	(0.687–0.800)
NLR	0.813	(0.759–0.868)

AUC calculated using the model predictions.

CI are estimated using DeLong’s method.

**Table 3:** Results of multivariate logistic regression for training cohort.

Characteristic	*N*	Event *N*	OR1	95% CI1	*p*-value
HCT	291	123	0.73	0.65, 0.81	<0.001
PCT	291	123	2.02	1.22, 3.35	0.006
APACHE-II	291	123	1.19	0.97, 1.45	0.088
NLR	291	123	0.90	0.82, 0.98	0.020

1OR, Odds Ratio; CI, Confidence Interval.

OR < 1 indicates a negative association between the predictor and IAI risk.

### 3.3 Predictive model development

**Figure 5:**
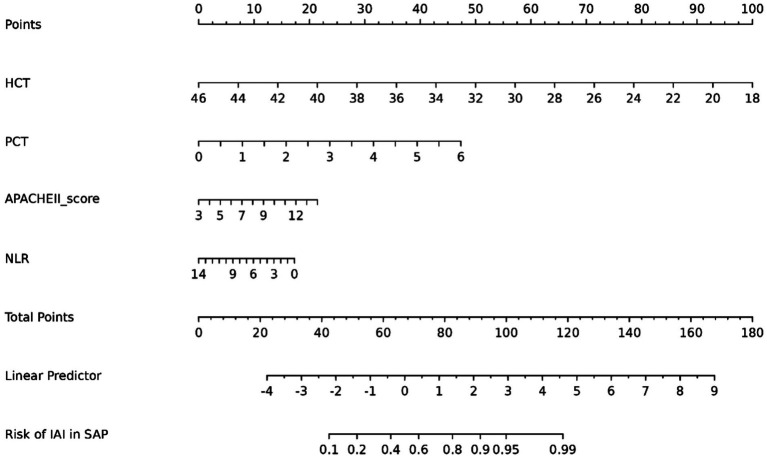
Nomogram for predicting IAI risk in SAP patients based on clinical risk factors.

Multivariable logistic regression confirmed PCT, APACHE II, NLR, and HCT as independent IAI predictors in SAP (Table 3). A clinical nomogram was constructed to quantify IAI risk stratification (Figure 5).

### 3.4 Predictive model validation

**Figure 6:**
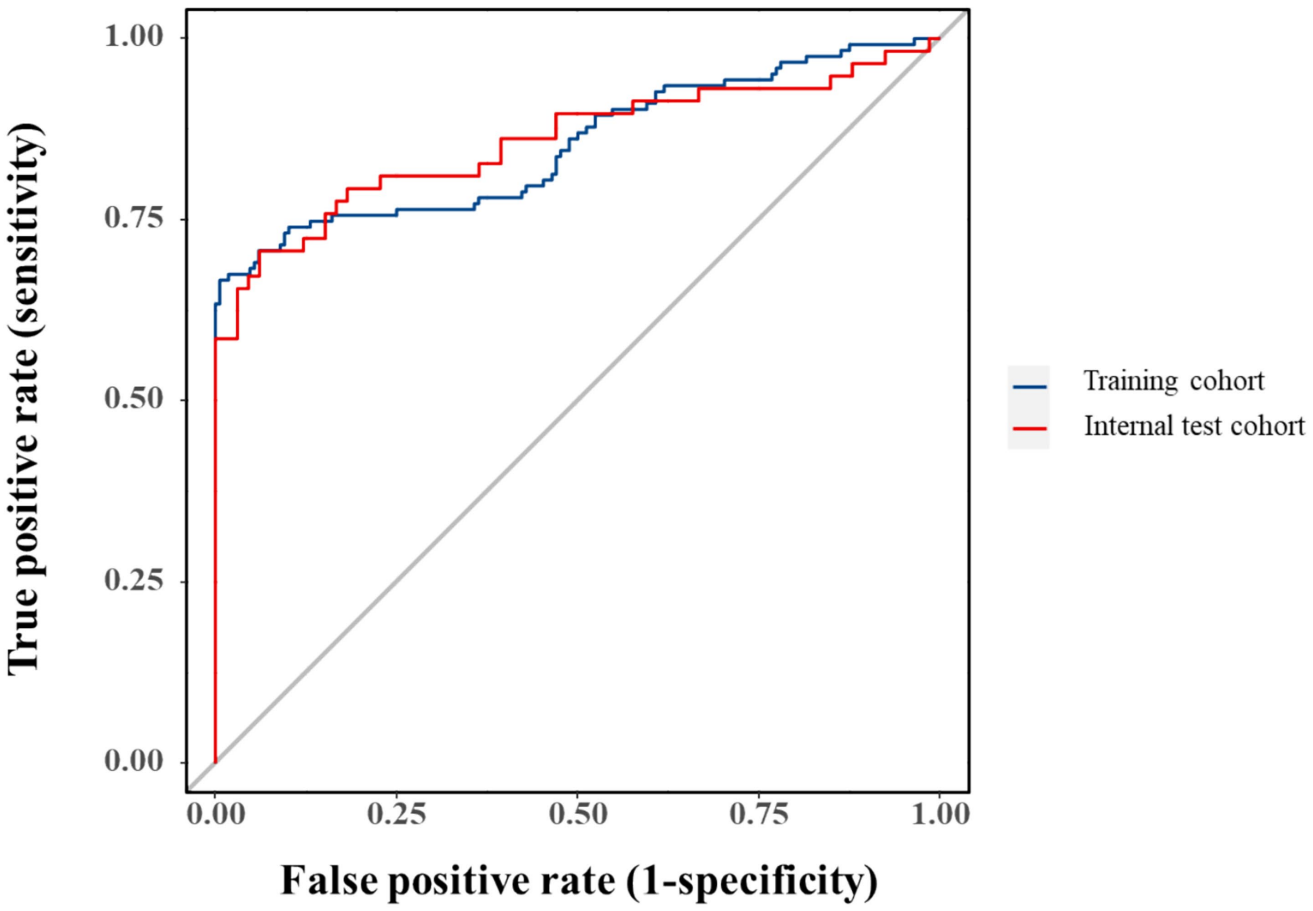
ROC curves for the predictive model in training and validation cohorts.

Model performance was robust in both training (AUC = 0.853, 95%CI:0.804–0.901) and validation cohorts (AUC = 0.858, 95%CI:0.786–0.930), though potential overfitting was considered given the comparable metrics (Figure 6). Bootstrap-corrected AUC (0.832) confirmed internal validity while underscoring the need for external validation.

**Figure 7:**
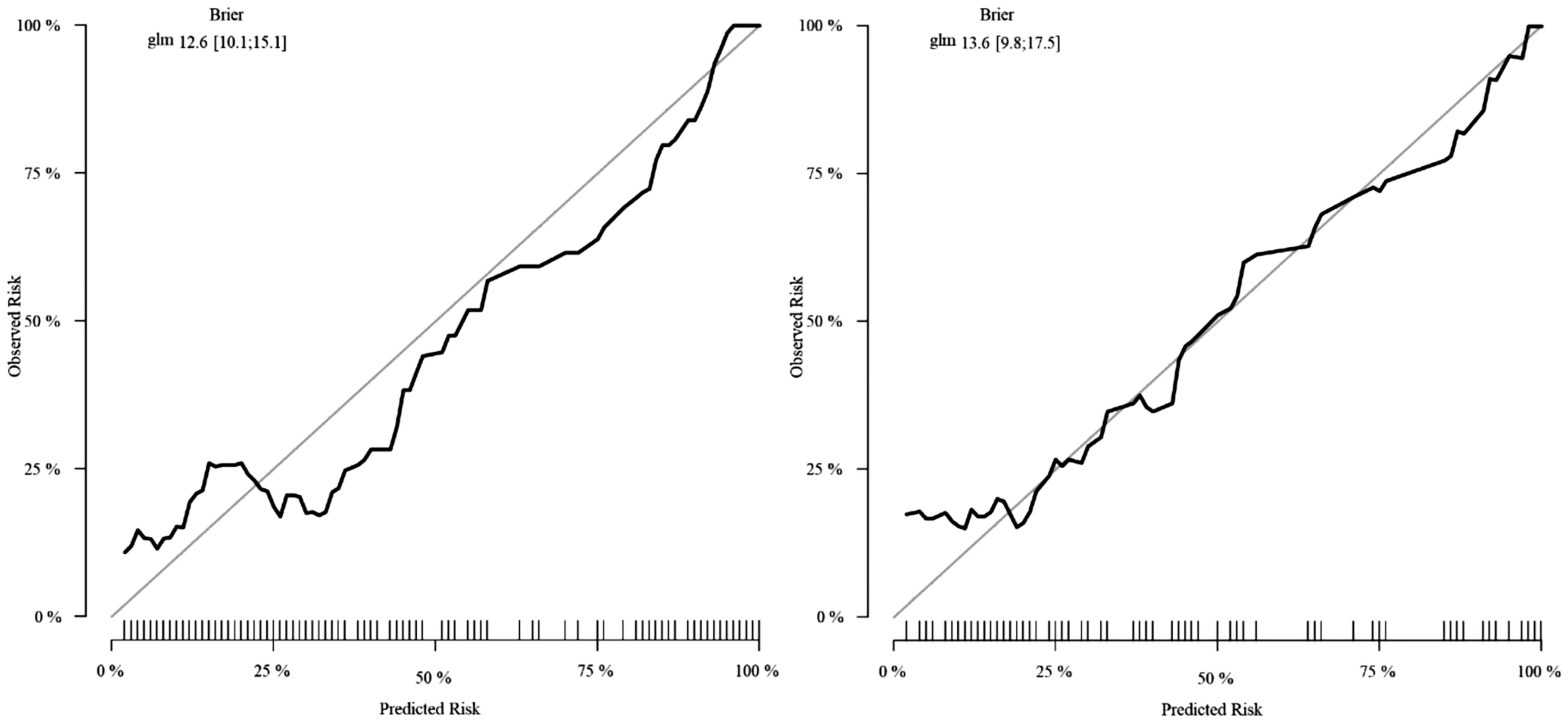
Calibration plots for the nomogram in training and validation cohorts.

**Figure 8:**
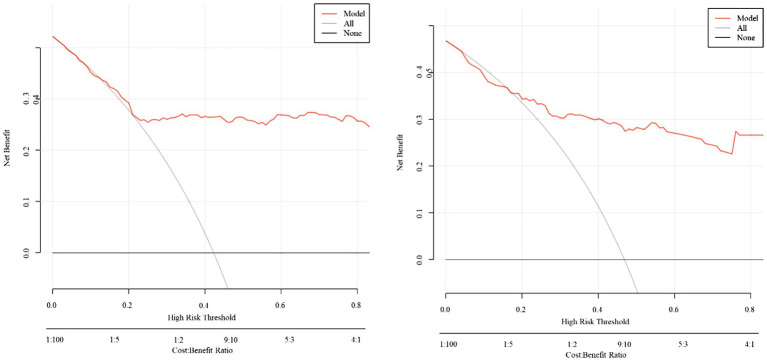
Decision curve analysis (DCA) for the predictive model.

Calibration analysis demonstrated excellent observed-predicted probability agreement across both cohorts (Figure 7). DCA revealed superior net benefit versus extreme strategies across clinically relevant threshold probabilities (Figure 8), supporting practical utility.

## 4 Discussion

IAI remains one of the most serious complications in patients with SAP, contributing significantly to morbidity and mortality. Early identification of high-risk patients is critical for timely intervention and improved outcomes. Among various prognostic scoring systems, the APACHE II score has been widely recognized for its utility in predicting disease severity and clinical outcomes in critical care settings, including neurosurgical and abdominal emergencies (9). Inflammatory biomarkers such as neutrophil CD64, IL-6, and PCT have also shown diagnostic value in infectious conditions like sepsis and may reflect the systemic inflammatory response that accompanies SAP with IAI (10). Furthermore, clinical profiling from tertiary care centers emphasizes the importance of integrating demographic characteristics, etiological factors, and laboratory indices to better stratify risk and guide management in acute pancreatitis (11). Based on these insights, we developed a clinical prediction model using multivariable logistic regression and presented it as a nomogram to facilitate individualized risk assessment for IAI in patients with SAP.

The analysis successfully identified four independent predictors of IAI in SAP—PCT, APACHE II, NLR, and HCT—through LASSO regression followed by multivariable logistic regression. The selection of these variables reflects a clinically plausible interplay between systemic inflammation (PCT, NLR), disease severity (APACHE II), and HCT in IAI pathogenesis. ROC analysis confirmed the individual discriminative capacity of each predictor (AUC > 0.5 for all variables), though their moderate standalone performance necessitated an integrated modeling approach for enhanced diagnostic precision.

The composite predictive model demonstrated robust discriminatory performance, achieving AUCs of 0.853 (95% CI: 0.804–0.901) and 0.858 (95% CI: 0.786–0.930) in the training and validation cohorts, respectively. These strong and consistent AUC values validate the model’s diagnostic utility while mitigating overfitting concerns through internal validation. Calibration analysis revealed excellent agreement between predicted probabilities and observed IAI incidence, with calibration curves closely approximating the ideal 45° reference line. This high-fidelity calibration, maintained across both cohorts, underscores the model’s reliability in risk stratification without significant bias.

DCA substantiates the model’s clinical utility by demonstrating superior net benefit across decision thresholds, suggesting its implementation could optimize diagnostic precision and resource utilization while improving patient outcomes. The model’s predictive accuracy supports its integration into clinical workflows for IAI risk stratification in SAP, facilitating timely interventions. By identifying high-risk patients requiring intensive monitoring or early empirical therapy, this approach may mitigate complications. The incorporation of routinely measured parameters aligns with precision medicine paradigms, enabling individualized risk assessment.

HCT represents an accessible and cost-effective hematological marker for pancreatitis severity assessment. However, its predictive performance exhibits interstudy variability. A large-scale investigation (*n* = 1,612) identified admission HCT and 24-h BUN dynamics as superior predictors of persistent organ failure and pancreatic necrosis compared to conventional parameters including APACHE II (12). Conversely, other studies have reported limited prognostic value of admission HCT in acute pancreatitis severity prediction (13).

PCT demonstrates significant diagnostic utility as a biomarker for severe bacterial/fungal infections, sepsis, and multi-organ dysfunction (14). Its discriminative capacity stems from pronounced elevation in systemic bacterial/fungal infections while remaining stable in viral or non-infectious inflammatory conditions (15, 16). In pancreatic pathology, PCT exhibits strong predictive value for infectious complications, with levels ≥3.8 ng/mL during days 3–4 post-onset demonstrating 79% sensitivity and 93% specificity for major complications (7). Notably, PCT reaches peak concentrations in cases of infected pancreatic necrosis with concomitant multi-organ failure. Meta-analytic evidence positions PCT as the most robust predictor of infected pancreatic necrosis (17), with its diagnostic accuracy further enhanced when combined with complementary markers like NLR and MCTSI (18, 19).

The APACHE II scoring system maintains established correlations with secondary pancreatic infections when integrated with inflammatory markers (20). The pathophysiological basis involves the inflammatory cascade in acute pancreatitis, characterized by proteolytic enzyme activation, cytokine release, and bacterial translocation. Neutrophil depletion correlates with favorable outcomes, whereas lymphocytosis associates with disease progression. NLR emerges as a clinically practical inflammatory index, providing real-time assessment of immune response dynamics through neutrophil-lymphocyte ratios, and has been validated as an independent predictor of pancreatitis severity (21).

The selected variables (PCT, APACHE II, NLR, and HCT) collectively capture critical pathophysiological dimensions of SAP-associated infection, including systemic inflammation, disease severity, and hematologic derangements. While individual ROC analysis confirmed each parameter’s discriminative capacity (AUC > 0.5), their modest standalone performance necessitated a multivariate approach to optimize diagnostic accuracy. The composite model achieved AUCs of 0.853 (training cohort) and 0.858 (validation cohort), with the minimal inter-cohort difference prompting consideration of potential over-optimism. Bootstrap validation (1,000 iterations) yielded an optimism-adjusted AUC of 0.832, supporting internal validity while underscoring the need for external validation in independent populations.

Compared with previous models, such as that by Zhu et al. (8)—who incorporated variables like intra-abdominal pressure, CTSI, and ICU admission to predict IAI—our model offers an alternative, laboratory-driven, non-imaging-based approach that may be more accessible in resource-limited settings. Unlike Zhu’s model, which emphasized radiological and procedural data, our model relies on routinely available biomarkers, facilitating earlier risk stratification upon admission. Nevertheless, their inclusion of imaging parameters may explain slightly higher discriminatory performance and should prompt future efforts to evaluate the incremental benefit of combining radiologic and laboratory predictors.

Additionally, Sun et al. (22) suggested that moderate platelet counts may serve as protective factors, and Qiu et al. (23) identified HCT as an independent risk factor for IAI—both findings aligned with our observations. However, we acknowledge that some biomarkers, such as CRP and IL-6, although commonly elevated in SAP, did not contribute incremental predictive value in our LASSO selection process—possibly due to collinearity with other variables or insufficient specificity. The model’s clinical implications lie in its simplicity, interpretability, and relevance to real-time clinical decisions. By integrating routinely available admission parameters into a nomogram, the model can support clinicians in identifying high-risk patients early (24, 25), prompting timely empirical antibiotic initiation, closer monitoring, or ICU triage.

The nomogram presented in this study offers a simple and precise method for predicting the risk of IAI in acute pancreatitis patients (26–28), which may improve survival rates during hospitalization.

Nonetheless, several limitations of this study should be acknowledged, as they may influence the interpretation and generalizability of the findings. First and foremost, the retrospective design inherently introduces potential selection and detection biases. Selection bias may arise from the inclusion criteria and clinical judgment used to enroll patients, while detection bias could result from non-uniform data collection or diagnostic practices. These biases may affect the observed associations and limit the internal validity of the study. Second, the study was conducted at a single center, which may limit the generalizability of the findings to other institutions or patient populations. Differences in clinical practices, patient demographics, and healthcare resources across centers could affect the model’s performance in diverse settings. Therefore, external validation using independent, multicenter datasets is essential to confirm the robustness and applicability of the predictive model. Future studies should aim to evaluate its performance across varied clinical environments to enhance its external validity and facilitate broader implementation. Third, key predictive parameters such as HCT, PCT, APACHE II score, and NLR are inherently dynamic and can vary substantially over time due to disease progression or clinical interventions. Relying on a single time-point measurement may not fully capture the evolving clinical status of patients with SAP, potentially limiting the model’s accuracy. Future studies should consider incorporating a dynamic, serial measurement approach to account for temporal trends and improve risk stratification. Such longitudinal tracking of biomarker trajectories may enhance the model’s clinical utility by providing real-time updates to risk assessments, thereby supporting more responsive and individualized patient management. Future studies should explore dynamic risk stratification using serial biomarker assessments and consider incorporating novel inflammatory or metabolic markers to improve predictive performance.

Although we adjusted for multiple known confounders using multivariable logistic regression, residual confounding due to unmeasured or unaccounted variables remains a notable limitation. Factors such as heterogeneity in clinical management, underlying comorbidities, and individual patient responses to treatment may have influenced outcomes but were not fully captured in our analysis. We recognize that these unadjusted variables could introduce bias into the model’s predictive performance. Future studies should aim to integrate a more comprehensive set of clinical, biochemical, and treatment-related parameters, potentially employing prospective designs or leveraging real-world data from EHRs, to improve confounding control and strengthen model validity.

Furthermore, in the training cohort, 90 patients developed IAI, resulting in an EPV ratio of 5.0 (based on 18 candidate predictors), which falls below the conventional threshold of 10. This limitation necessitates cautious interpretation of the model’s results. While internal validation was performed, external validation using an independent dataset from a tertiary care center is planned to evaluate the model’s generalizability and robustness across diverse clinical environments. The relatively low EPV also raises concerns regarding potential overfitting and diminished model stability. Although variable selection techniques were implemented to mitigate this risk, we explicitly acknowledge this constraint. Subsequent research should prioritize larger sample sizes with sufficient outcome events or incorporate penalized regression approaches to enhance generalizability. Additionally, *post hoc* power calculations and external validation in independent cohorts are warranted to further assess the reliability of our findings.

## 5 Conclusion

The predictive model incorporating HCT, PCT, APACHE II score, and NLR demonstrates strong discriminatory accuracy in stratifying IAI risk among SAP patients, offering a valuable tool to optimize clinical decision-making and improve patient outcomes.

## Data Availability

The raw data supporting the conclusions of this article will be made available by the authors, without undue reservation.

## Footnotes

^1^ http://www.mstata.com
