# Supplementary material for: Development of a clinical prediction model for intra-abdominal infection in severe acute pancreatitis using logistic regression and nomogram
Source: Front Med (Lausanne). 2025 Aug 7;12:1636733. doi: 10.3389/fmed.2025.1636733 (PMC12367684; doi:10.3389/fmed.2025.1636733)
Supplement: Supplementary file 1 [file Table_1.docx]

Table S1. Variance Inflation Factors (VIFs) for Predictors in Final Model

| **Variable** | **VIF** |
| --- | --- |
| HCT | 1.21 |
| PCT | 1.12 |
| APACHE II | 1.45 |
| NLR | 1.38 |

Note: All VIFs < 2, indicating low multicollinearity.
